# Genes for Yield Stability in Tomatoes

**DOI:** 10.1002/ggn2.202100049

**Published:** 2021-12-09

**Authors:** Josef Fisher, Dani Zamir

**Affiliations:** ^1^ The Institute of Plant Sciences Faculty of Agriculture The Hebrew University of Jerusalem PO Box 12 Rehovot 76100 Israel

**Keywords:** breeding, canalization, DnaJ chaperon, QTL, *Solanum lycopersicum*, yield stability

## Abstract

Breeding plant varieties with adaptation to unstable environments requires some knowledge about the genetic control of yield stability. To further this goal, a meta‐analysis of 12 years of field harvest data of 76 *Solanum pennellii* introgression lines (ILs) is conducted. Five quantitative trait loci (QTL) affecting yield stability are mapped; IL10‐2‐2 is unique as this introgression improved yield stability without affecting mean yield both in the historic data and in four years of field validations. Another dimension of the stability question is which genes when perturbed affect yield stability. For this the authors tested in the field 48 morphological mutants and found one ‘canalization’ mutant (*canal‐1*) with a consistent effect of reducing the stability of a bouquet of traits including leaf variegation, plant size and yield. *canal‐1* mapped to a DNAJ chaperone gene (Solyc01g108200) whose homologues in *C. elegans* regulate phenotypic canalization. Additional alleles of *canal‐1* are generated using CRISPR/CAS9 and the resulting seedlings have uniform variegation suggesting that only specific changes in *canal‐1* can lead to unstable variegation and yield instability. The identification of IL10‐2‐2 demonstrates the value of historical phenotypic data for discovering genes for stability. It is also shown that a green‐fruited wild species is a source of QTL to improve tomato yield stability.

## Graphical abstract

Breeding tomatoes for stable yield. Using 12 years of field data, five quantitative trait loci that affect the variation in fruit yield are found in introgression lines from wild tomato. One QTL stabilizes yield without affecting mean yield. In contrast, a screen for morphological mutants in production tomato lines found one locus that reduces stability in fruit yield, leaf variegation, and plant size.

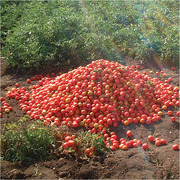

## 1 Introduction

To maximize crop productivity, farmers select for cultivation those varieties that produce high yields in diverse agricultural environments.^[^ ^1^, ^2^ ^]^ For this reason, new experimental varieties are being tested in replicated field trials that are conducted in different locations, with the most stable performers, with the highest yields, being recommended for commercial cultivation. The datasets generated by past variety trials harbor a trove of information for the future identification of quantitative trait loci (QTL) and genes that regulate stability of agricultural performance.

From the plant breeding and the biological points of view, considerably more is known about genes that modify the means than about genes that affect its dispersion. The ability of a crop variety to perform in a consistent manner can be attributed to simple genetic factors such as susceptibility to a particular disease that is prevalent in certain sites and not others. In such a case, the yield instability can be simply be overcome by introducing a single resistance gene. Beyond such obvious cases the search is on for the so called ‘master‐regulators’ that buffer traits against genetic and environmental perturbations by modulating genetic networks.^[^ ^3^, ^4^ ^]^ The best‐known example of a master regulator is the molecular chaperone *HSP90*, which assists in the folding of many proteins. Although there is still some ambiguity regarding the role and mode of action of the chaperone *HSP90* it is tempting to speculate that among the many “genes of unknown function” there exist additional factors that act on the biological system as a whole.^[^ ^5^ ^]^ Support for this comes from a mutant screen for master regulators in *Saccharomyces cerevisiae* that showed more than 300 gene products that when disrupted led to increased morphological variation.^[^ ^6^ ^]^

To identify genes that may act as master regulators we screened in the field tomato diversity of wild species introgression lines (ILs) in a genetic background of a cultivated inbred variety M82 and also induced mutations in the same inbred processing tomato variety. To phenotype the tomato entries for yield stability we applied a ‘canalization replication’ (CANAREP) design where each such replication consisted of eight plants of identical genotypes (in a row), where yield of each of the plant was used to estimate the mean and the standard deviation that was used to calculate the coefficient of variation (CV = standard deviation/mean) for the canalization replication.^[^ ^7^ ^]^ CANAREP estimated CV for multiple traits generated values that could be statistically compare between genotypes. The advantage of the CANAREP is that the estimation of stability is robust but at a price of a ten‐fold increase of the number of plants in the experiments.

## 2 Results

### 2.1 Wild Species ILs that Modify Yield Stability of a Cultivated Variety

**Figure 1:**
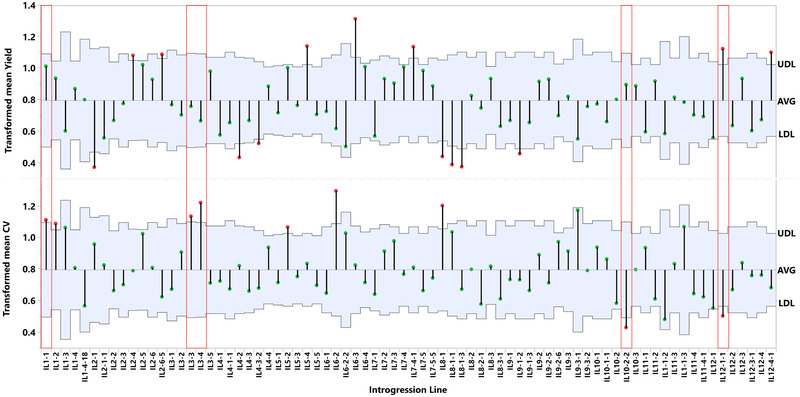
Yield related traits metanalysis of 12 years of introgression line (IL) field trials. The IL population is ordered by their names from left to right. For each trial the IL mean value of total fruit yield was compared to M82 at the same season. A) The total yield of each IL compared to M82. B) Presents the differences in coefficient of variation (CV) of total yield compared to M82. The ILs marked by red boxes were selected for further research (i.e., IL1‐1, 3‐3, 3–4, 10‐2‐2, 12‐1‐1). Points that lie outside the upper decision limit (UDL) or lower decision limit (LDL) are statistically different from the grand mean using an alpha = 0.05.

The first genetic resource we screened to identify yield stability QTL was the *S. pennellii* (LA716) IL population, for which historical data on yield associated traits was available from replicated trials conducted over 12 years^[^ ^8^ ^]^ (DRYAD). We observed that different ILs that share a common *S. pennellii* genomic segment often exhibited similar phenotypic effects on yield and its stability (**Figure** **1**): The overlapping pairs IL3‐3/IL3‐4 and IL1‐1/IL1‐2 exhibited a large dispersion of mean yield (high CV) in the historic data. The overlapping pairs IL10‐2/IL10‐2‐2 and IL12‐1/IL12‐1‐1 produced in the historical data mean yields similar to M82, with lower CVs relative to the cultivated variety, that is, these wild species genomic regions conferred higher yield stability.

**Figure 2:**
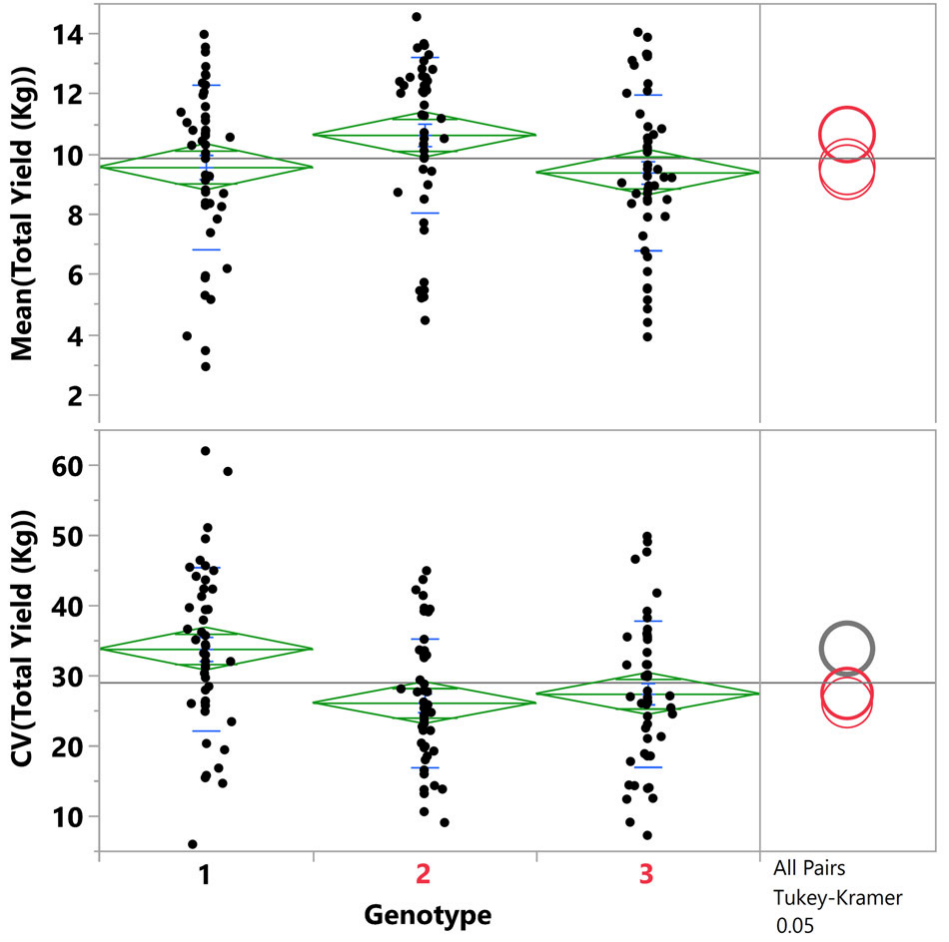
Four seasons summary of IL10‐2‐2 field trials. Each dot in this figure represents the mean total yield and the mean coefficient of variation (CV) of total yield of a single CANAREP, measured for four years (46 CANAREPS for each line). A) A comparison between the three genotypes monitored (1 = M82, 2 = heterozygous, 3 = IL10‐2‐2) for total yield (TY); B) Coefficient of variation (CV) of total yield comparison for the same plots measured in A. The statistic comparisons were performed using the Turkey–Kramer test with an alpha = 0.05 and the comparison circles of different colors indicate a significant difference; the green diamond indicates the mean of the studied genotypes. This figure emphasizes the QTL effect of IL10‐2‐2, as having a canalization effect with only a small influence on mean yield values.

In 2013 we started the validation of stability using CANAREPS of IL1‐1, IL3‐3, IL3‐4, IL12‐1‐1 (Table S1, Supporting Information), and IL10‐2‐2 (**Figure** **2**). All the ILs maintained the trend of their yield stability effects except for IL12‐1‐1 that did not stabilize yield more than the isogenic control. In the following three years, the canalization trials focused on IL10‐2‐2 using as a seed source the bulks described in the Section 4 to ensure that residual unlinked and/or undetected introgression(s) in the original IL10‐2‐2 were actually those which affected the stability phenotype. Figure 2 shows the combined analysis of IL10‐2‐2 over four years of testing, which included 46 canalization replications (1273 plants altogether) that were measured for yield traits (total yield, plant weight, fruit weight, brix).

Finer mapping of the yield stability QTL associated a smaller 470 kb genomic region with the phenotype (Figure S1, Supporting Information). It is important to note that IL10‐2‐2 only had an effect on stability yield. Additionally, the effect of the *S. pennellii* genomic segment on stability was limited to total yield per plant and did not affect the level of stability of other morphological traits that were measured, such as fruit weight or Brix (Table S2, Supporting Information).

### 2.2 Mutants Affecting Yield Stability

**Figure 3:**
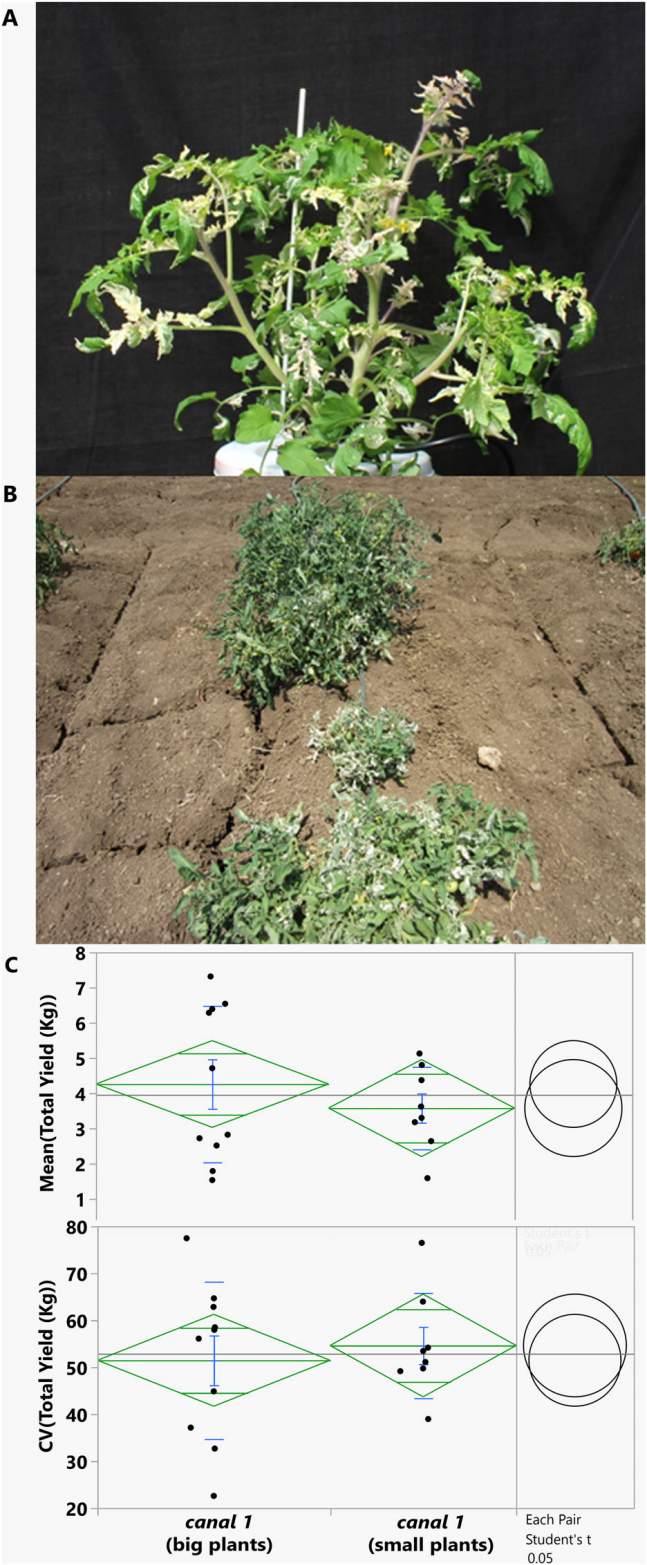
The *canal‐1* mutant. A) Picture of a plant mutated in the gene Solyc10g108200 (*canal‐1* ^e4058^). B) A typical CANAREP of *canal‐1* ^e4058^ showing plants that vary in their sizes. C) Measurement of progeny of large and small plants for total yield and the coefficient of variation (CV) of total yield. The statistical comparison was done using Student's test at an alpha level of 0.05. Within the mean diamond are the standard deviation bars above and below the mean of each group (in blue). This figure shows that there is no difference in the total yield phenotype of progenies of plants of different sizes, which means that the mutant itself is responsible for the unstable phenotype.

To identify genes that when disrupted alter yield stability we screened in canalization replications 48 M82 mutants.^[^ ^9^ ^]^ Each homozygous mutant was grown in three normally irrigated CANAREPs and three in dry ones; Table S3, Supporting Information lists the mutants, sorted by yield stability CV. The lowest stability was found for the mutant e4058, which uniformly had variegated leaves and was later named *canalized‐1* (*canal‐1*; **Figure** **3**A). This instability could be easily seen in the field within the canalization replications with very small plants while others were M82‐size (Figure 3B). This raised the possibility that another mutation that affects plant size segregated in the F5 lines. To test this possibility, selfed seed from five small plants and five large plants were collected and planted in the field in CANAREPS ‐ no differences were found between the means and CVs of the progeny of the large versus small plants (Figure 3C). If the existence of small plants was due to another mutation that segregated in the line, we would have expected all the progeny of the small plants to be small. Thus, we can conclude This shows that plants homozygous for the *canal‐1* ^e4058^ allele can exhibit markedly different phenotypes in the field, which is the hallmark of canalization genes where a single genotype can produce multiple phenotypes.

To genetically map the variegation effect of *canal‐1* ^e4058^, a mutant plant was crossed with the red‐fruited wild species *S. pimpinellifolium* (LA1589) and in the F2 we counted 48 plants with no‐variegation and 19 variegated ones. The preliminary mapping of *canal‐1* to *chromosome‐1* was achieved using DNA from the above F2 plants using an indel markers kit covering the entire genome. Higher‐resolution mapping was performed on an additional 120 variegated F2 plants, which localized *canal‐1* to an interval of 210 000 base pairs that included two dozen genes. Among them was Solyc01g108200, a homologue of At3g19220, that was identified as *cyo‐1* and later named *Snowy Cotyledon 2* (*SCO2*).^[^ ^10^, ^11^ ^]^

The unique characteristics of the tomato *canal‐1* prompted us to search for additional alleles by crossing *canal‐1* ^e4058^ with different variegated accessions. The mutant *ghost‐2* (*gh‐2*; LA2007) from the Tomato Genetics Resource Center (TGRC, UC Davis) was found to be allelic to *canal‐1* ^e4058^, while the mutants e1458, e9515, and a Core Collection variety (CC1300) did not exhibit variegation in their respective F1s with *canal‐1* ^e4058^ and thus were considered non‐allelic. The F1 hybrids of *canal‐1* and *gh‐2* were very stable in their drastic variegation which was higher than their homozygous parent. The best candidate for being *canal‐1* was Solyc01g108200.2 (Solanaceae Genome Network https://solgenomics.net/) which is 3600 base pairs‐long and composed of 3 exons, which are translated to a protein of 203 amino acids. Both alleles result from mutations in the second exon; *canal‐1* ^e4058^ was shown to have a T→G (W→G) substitution 923 bp downstream of the transcription start site, while the mutation of *gh‐2* was delimited to a C→T (P→L) substitution 1011 bp downstream of the transcription start. A CAPS marker for *gh‐2* designed to distinguish between M82 and *gh‐2* alleles, co‐segregated perfectly with the phenotype of variegated leaves of 100 plants. Targeted editing of the first exon of Solyc01g108200 using CRISPR/Cas9, led to identification of two sterile mutant plants. The first (named *CR9*) germinated well but had white cotyledons and was unable to form true leaves (Figure S2D, Supporting Information). This genotype was found to have a deletion of six bases (Figure S2A, Supporting Information) leading to a premature stop codon after only 21 amino acids (Figure S2C, Supporting Information). The second genome edited line (CR6.1, Figure S2E, Supporting Information) was found to have a deletion and an insertion of a single nucleotide (Figure S2B, Supporting Information), generating a frame shift of 29 amino acids, from amino acid 15 to amino acid 43 (Figure S2C, Supporting Information). These results suggest that only specific mutations in Solyc01g108200 can cause the instability of the phenotype, while other perturbations in the gene create variegated phenotypes that are uniform for all plants of a particular genotype.

## 3 Discussion

Geneticists know considerably more about specific genes that affect the mean value of traits than about those that control the degree of dispersion of the means. The latter stability trait is particularly important for agriculture where we aim grow varieties that are relatively stable in their good productivity also in climate fluctuations. A common parameter that is used to estimate yield stability is the coefficient in linear regression that describe the performance of genotypes across different trial environments. The deviation from the estimated regression describes a ‘stability index’ which can be used to compare yield consistency of different genotypes. Regression analysis of the stability index on polymorphic genetic markers has been performed to map candidate genomic regions that are associated with yield stability in barley,^[^ ^12^ ^]^ soybean,^[^ ^13^ ^]^ sunflower,^[^ ^14^ ^]^ wheat,^[^ ^15^ ^]^ and tomato.^[^ ^16^ ^]^ In our study we describe the screening, identification, mapping and validation of genomic regions and a gene that affect yield stability in tomato. This was achieved using canalization replication (CANAREP) experimental design that enables the measurement of yield associated traits for each member of a group of eight plants at equidistance along the row in the field. Using this approach, we can estimate the diversity of plants that are exposed to a similar field environment in a manner that allows the calculation of the mean, standard deviation and the CV (ratio of standard_deviation/mean) for each replication unit. When multiple such units are used for a number of genotypes the CV for yield can be statistically compared and the most stable and unstable lines can be selected.

The first tomato genetics resource we used to map stability QTL was the *S. pennellii* introgression lines. This set of fixed genotypes^[^ ^8^ ^]^ was phenotyped in the field, with multiple replicates per trial, over a 12 year period. This database allowed us to identify specific QTL that based on the historic data altered CV of yield associated traits. The objective of the 12 years of trials was to identify and validate QTL that affect the means of traits while in this paper, we use the data to analyze the stability of the phenotype over years. To validate the historic data, we established canalization replications trials with 15–20 such CANAREPS for each genotype. The two unstable genomic regions IL1‐2, and the overlapping ILs that share a common genomic region on chromosome 3 (IL3‐3/IL3‐4) were easily validated as the phenotype was altered for a bouquet of correlated traits; additionally, the mean yield of lines homozygous for these segments were dramatically reduced compared to the control M82. The stable pairs IL10‐2‐2 and IL12‐1‐1 both had a similar mean yield as the control M82 but only IL10‐2‐2 validated in our canalization replication set‐up. It is possible that we could not detect a yield stability effect for IL12‐1‐1 due to the experimental set‐up: in the historical trials the entire IL collection was grown in a randomized manner in the experimental plot while the validation was conducted using CANAREPS where the neighbors of each plant were others of the same genotype. To eliminate the possibility that the yield stability phenotype is not due to IL10‐2‐2 we derived every year different stocks from self‐pollination of heterozygotes followed by marker analysis and field planting. Since the tomato variety M82 has been one of the top producers in the pre‐hybrid era of processing tomatoes it initially seemed surprising that a genomic segment of *S. pennellii* could improve yield stability of a very successful variety. On the other hand, yield in tomatoes correlates to fruit number and so is reproductive success in nature. Thus, it makes sense that some genes that confer selective advantage in natural habitats could also stabilize production in agricultural fields. Finally, this IL analysis is an example for the potential value of sharing the raw phenotypic data with the community.^[^ ^17^ ^]^ When scientists involved in plant genetics research deposit the raw data in public databases it could be analyzed in the future to probe other biological aspects that were not addressed in the original paper.

The genetic basis of the buffering capacity of the organismal phenotype against environmental and genetic perturbations (canalization) was investigated in yeast, in a study which quantitatively phenotyped the budding of 4718 viable single‐gene deletions in haploid strains.^[^ ^6^ ^]^ This landmark study identified 300 genes that, when deleted, result in an increase of the morphological variation and were therefore defined as “phenotypic capacitors”. Among these capacitors, there was an overrepresentation of genes that are involved in essential cellular processes, such as maintaining DNA stability, RNA processing and cell cycle. The yeast screen suggested that ≈5% of the genes are involved in buffering the phenotype against environmental and genetic perturbations.

These results prompted us to screen for yield stability a subset of 48 single gene mutants derived from a large mutagenesis project of the variety M82.^[^ ^9^ ^]^ Of the 48 mutations tested the most pronounced effect on yield stability was observed for the e4058 allele of SOLYC01G108200.2, a tomato homologue of the *Arabidopsis thaliana* mutant *snowy cotyledons‐2* (*SCO2*, At3319220). The Arabidopsis gene encodes a DNAJ‐like protein, with a zinc‐finger domain that has a chaperone activity and is involved in thylakoid biogenesis.^[^ ^18^ ^]^ *SCO2* is required for normal assembly of the photosynthetic machinery in cotyledons and possibly, for the targeting of the light harvesting chlorophyll‐binding protein LHCB1 to the thylakoid membrane. While the mutant was initially thought to be cotyledon‐specific, it was recently shown that under short‐day conditions, the true leaves could also become variegated.^[^ ^19^ ^]^ Over four years of field studies, the tomato *canal‐1* ^e4058^ mutant showed an unstable phenotype, that is, plants of identical genotypes homozygous for the mutation showed large variation in plant size, leaf variegation and yield. Similarly, a *C. elegans* study showed that seven of eight endoplasmic reticulum associated DNAJ genes are involved in trait canalization.^[^ ^20^ ^]^ It is important to note that an independent allele at the same locus (*ghost‐2*), as well as two CRISPR/CAS9‐generated mutations had leaf variegation but did not exhibit the unstable variegation phenotype. *ghost‐2* produced uniformly large plants and the CRISPR/CAS9 mutants did not survive beyond the first true leaf stage. This result is consistent with other studies that showed that independent mutations in the same gene may result in different phenotypes.^[^ ^21^ ^]^ The instability of *canal‐1* ^e4058^ that is located on chromosome 1 (Genome coordinates: SL2.50ch01:95554613‐95560134) suggests that the site of the mutation in the second exon (T→G (W→G) substitution, situated 923 bp downstream of the transcription start site, has a function associated with the stability of the phenotype. However, we did not explore the molecular function of *canal‐1* ^e4058^ and this must await additional investigations.

While *canal‐1* ^e4058^ reduced yield stability, another locus on IL10‐2‐2 improved yield stability of a tomato variety without a major effect on the mean yield. Wild species are selected in nature to survive and reproduce in diverse environments, whereas crop plants rely on human agronomic care. Here we show that natural biodiversity can be a source of genes and QTLs to improve yield stability even when benchmarked to an elite processing tomato variety.

## 4 Experimental Section

### Mining of Introgression Lines Historical Data and a Phenotyped Mutant Resource

*Introgression Lines*: To identify QTL that affect the stability of yield associated traits, the authors used the introgression lines (IL) population that covered the entire genome of *Solanum pennellii* in 76 overlapping segments in the genetic background of domesticated processing tomato (*S. lycopersicum*) variety M82.^[^ ^22^ ^]^ Because the ILs differ from each other by only a single *S. pennellii* chromosome segment, they are extremely useful for identification of QTLs, since any phenotypic difference between an IL and the recurrent parent M82 can be attributed to the introgressed chromosome segment.

Between the years 1993 and 2004, the *S. pennellii* ILs were phenotyped on multiple occasions in a homozygous and heterozygous condition in replicated field trials conducted at the Akko experiment station.^[^ ^8^ ^]^ In each year IL 6–12 individual plants per IL were randomized in the field (1 plant per m^2^) and were measured for the yield‐associated traits. This enabled for every trait to calculate a single CV estimate per IL, per year. The estimates from 12 years of trialing and the SNP genotypes of the ILs allowed the authors to compare trait stability (CV) among the lines and to map putative wild species genomic regions affecting this dispersion parameter.^[^ ^7^ ^]^ By normalizing the results of each season relative to the common control M82 we selected based on the historic data five lines with the most stable and unstable yield associated phenotypes for validation.

*Mutants*: The second genetic resource that was used in our search for stability genes was an M82 mutant collection generated by chemical and physical means that is called “The Genes that Make Tomatoes” that includes 3500 phenotyped hits.^[^ ^9^ ^]^ 48 mutants from the collection with effects on a range of traits compared to the isogenic control M82 was selected (Table S3, Supporting Information).

The ILs and the mutants were subjected to a similar yield stability tests in the field. All seed were sown in the Histill nursery, Ashkelon, in early March and were transplanted in canalization replications in mid‐April. At maturity (90–100% ripe fruits in the field), the following phenotypes relating to total yield were measured: plant weight (PW kg), total yield (TY kg), harvest index (HI), fruit weight (FW g) and Brix (% total soluble solids in the fruit).^[^ ^23^ ^]^

### Field Validation of Historical IL Data and the Isogenic Mutants

Five ILs (IL1‐1, IL3‐3, IL3‐4, IL10‐2‐2, IL12‐1‐1) that affected yield stability were tested in spring season of 2013 in Akko Experiment Station. Each IL was grown in five ‘canalization replications’ under optimal irrigation conditions (≈300 m^3^ of water per 1000 m^2^) and in five canalization replications under drought stress (≈40 m^3^ of water per 1000 m^2^ applied immediately after transplantation). Each replication was composed of 10 plants of the same genotype, planted at equal spacing (50 cm between plants) where the eight middle plants were measured for morphological and yield‐associated traits and the CV for the entire group was calculated. To eliminate the possibility that other undetected genetic variations contributed to the observed results, F2 seeds resulting from selfing of the respective IL hybrids (ILHs) were planted. In the nursery the F2 seedlings were separated to the three genotypic groups using PCR markers for the targeted wild genome introgression (Table S4, Supporting Information): *1*‐homozygous for the cultivated tomato allele, *3*‐ homozygous for the *S. pennellii* allele, and *2*‐ heterozygotes. After the genotyping, the seedlings of each group were tagged and transplanted in canalization replications and the seed collected from the genotypes *1* and *3* constituted the seed source for the coming trials.

The 48 mutants were initially screened using three canalization replications grown in optimal irrigation conditions and three grown under drought stress (i.e., irrigated with 20% of water compared to optimal conditions). Evaluation of trait dispersion was based on the mean CV of the six plots that included the same mutant; no difference in the CV was observed between the wet and the dry treatments.^[^ ^7^ ^]^ An in‐depth investigation of the selected unstable mutant e4058 (*canal‐1*) was conducted in four different seasons.

### Fine Mapping of ILs and Mutants

To further the understanding of the QTL for yield stability (IL10‐2‐2 spans ≈1.7 million base pairs) and the mutant *canal‐1*, a strategy of finer mapping was followed. Five indel markers were designed for the introgressed region of IL10‐2‐2 by comparing of the DNA sequence of *S. pennellii* (LA716) and M82. The indels were validated by amplifying the flanking regions using Primer 3 program (http://bioinfo.ut.ee/primer3‐0.4.0/) (Table S5, Supporting Information). The indel markers were used to establish subpopulations of different introgressed regions of IL10‐2‐2 and there were used to map the stability effect.

The variegated mutant, *canal‐1^e4058^*, in the genetic background of M82, was fine mapped in an F2 family from the red fruited wild species *Solanum pimpinellifolium* (LA1589). The F2 population was screened for individuals homozygous for the mutation that were used for mapping of a genomic region that does not have the *S. pimpinellifolium* polymorphism since all plant that were homozygous for *canal‐1^e4058^* mutant carried only the cultivated tomato alleles in the vicinity of the mutation. Using a set of 48 indel markers, designed by exploiting the natural genomic diversity between the red tomato ancestor *S. pimpinellifolium* and the cultivated tomato *S. lycopersicum*, M82. The 48 indel markers were distributed over all tomato chromosomes.

Additional markers were designed to fine map the mutated gene. Selected genomic regions showing high diversity between the genomes of *S. pimpinellifolium*, and *S. lycopersicum*, were compared and aligned. When a polymorphic site was identified, primers were designed for the flanking region. When a single nucleotide polymorphism (SNP) site was identified, a dCAPS designer tool^[^ ^24^ ^]^ was used to identify restriction enzymes that recognize the polymorphic site. A DNA sequence for the candidate gene Solyc01g108200 was prepared at The Center for Genomic Technologies at the Hebrew University of Jerusalem using the Sanger DNA sequencing method.

### Generating CRISPR/Cas9 Mutations in Solyc01g108200

The protocol of Brooks et al.^[^ ^25^ ^]^ with two guided mRNA sequences primers, was used for the CRISPR/Cas9 gene editing of Solyc01g108200 (*canal‐1*):

CR‐CANAL1 F1 tgtggtctcaATTggaagtaatgaagaacttaggttttagagctagaaatagcaag

CR‐CANAL1 F2 gtggtctcaATTgaaccaccagcgaaaggacggttttagagctagaaatagcaag

M82 was the genetic background that was selected for this gene editing activity. DNA from mutant plants showing the expected phenotype were sequenced using the flanking primers:

5’‐ TTGGTCCTATCTCCGTCCAT‐3’

5’‐ TACCCTGACACTTCCCCTTG‐3’

## Conflict of Interest

The authors declare no conflict of interest.

## Author Contributions

D.Z. conceived the research plans; J.F. performed the experiments; D.Z. and J.F. wrote the paper.

**Data table IL historical data** contains phenotypes of yield related traits in the *S. pennellii* (LA716) IL population, from replicated trials conducted over 12 years 1993–2004.

**Data table Akko 13** contains detailed phenotyping for five ILs (IL1‐1, IL3‐3, IL3‐4, IL10‐2‐2, IL12‐1‐1) that affected yield stability tested in spring season of 2013 in Akko Experiment Station.

**Data table 10‐2‐2 Akko 13–17 raw CANAREPS** contains detailed phenotyping for CANAREPS experiments on IL10‐2‐2 shown in Figure 2.

## Supporting information

Supporting Information

## Data Availability

Data was deposited in Dryad. https://datadryad.org/stash/share/0WafQT4zMdUwBaR6gsNcUPQS2TKkHAmkUYYlkCNDETg
