# Supplementary material for: Genes for Yield Stability in Tomatoes
Source: Adv Genet (Hoboken). 2021 Dec 9;2(4):2100049. doi: 10.1002/ggn2.202100049 (PMC9744526; doi:10.1002/ggn2.202100049)
Supplement: Supplementary file 1 — Supporting Information [file GGN2-2-2100049-s001.pdf]

Supplemental information titles and legends

Supplemental Figure 1

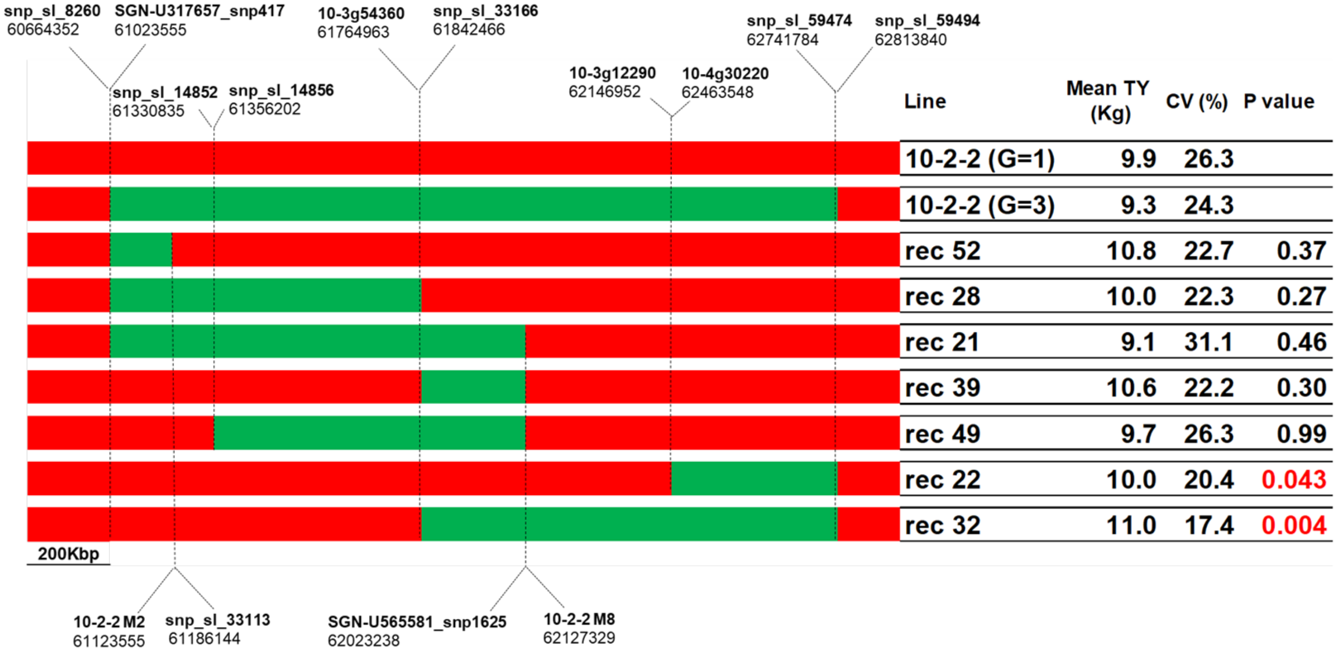

Line	Mean TY (Kg)	CV (%)	P value
10-2-2 (G=1)	9.9	26.3	
10-2-2 (G=3)	9.3	24.3	
rec 52	10.8	22.7	0.37
rec 28	10.0	22.3	0.27
rec 21	9.1	31.1	0.46
rec 39	10.6	22.2	0.30
rec 49	9.7	26.3	0.99
rec 22	10.0	20.4	0.043
rec 32	11.0	17.4	0.004

Supplemental Figure 1. Sublines of IL10-2-2. This figure shows the selected recombinants of IL10-2-2. The first red bar represents the cultivated genome marked as 10-2-2 (G=1). The second bar, colored in green, represents IL 10-2-2. The two markers that defined the break point between the cultivated and the wild genomes are shown at the break point. The name of the marker is indicated in bold and the genomic position on chromosome 10 is indicated under the marker name. On the right side of the figure, mean total yield (TY) and mean coefficient of variation (CV) for the different lines, and t test analysis of the CV values of each recombinant as compared to M82 (i.e., 10-2-2 (G=1)), are listed. Two recombinants showed a significant difference (rec 32, rec 22) in their CV values. The two shared a *S. pennellii* segment, which contains about 44 genes with none of the usual suspects.

Supplemental Figure 2

A

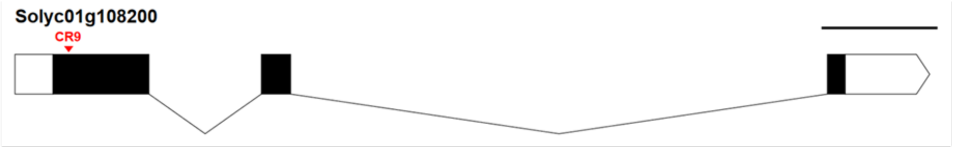

M-82	TTGAACCACCAGCGAAAGGACGAGGGTCCGC	M-82	AGGAAGTAATGAAGAACTTAGAGGAGTGA
gRNA	TTGAACCACCAGCGAAAGGACGAGGGTCCGC (PAM)	gRNA	AGGAAGTAATGAAGAACTTAGAGGAGTGA (PAM)
CR9	TTGAACCNCCAGCGAA---- CGAGGGTCCGC	CR9	AGGAAGTAATGAAGAAC--AGAGGAGTGA

B

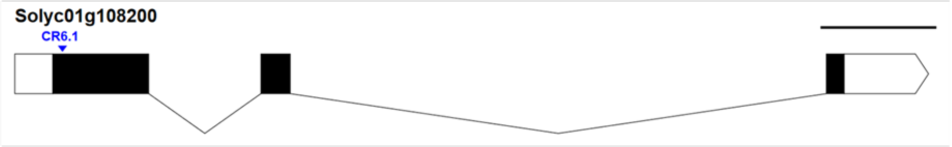

M-82	TTGAACCACCAGCGAAAGG ACGAGGGTCCGC	M-82	AGGAAGTAATGAAGAACTTAGAGGAGTGA
gRNA	TTGAACCACCAGCGAAAGG ACGAGGGTCCGC (PAM)	gRNA	AGGAAGTAATGAAGAACTTAGAGGAGTGA (PAM)
CR6.1	TTGAACCACCAGCGAAAGGGACGAGGGTCCGC	CR6.1	AGGAAGTAATGAAGAAC- TAGAGGAGTGA

C

M-82	MLPLINSCSIFTPLSSSLLPPRRSTVISCQAAADPSAAGGPSSFRWAAAAPGFG/ /DLDDKPP-
CR6.1	MLPLINSCSIFTPLVLHYFLLVDPPSLVAKPLLTHQLPADPRPFRWAAAAPGFG/ /DLDDKPP-
CR9	MLPLINSCSIFTPLFFITSSS-

D

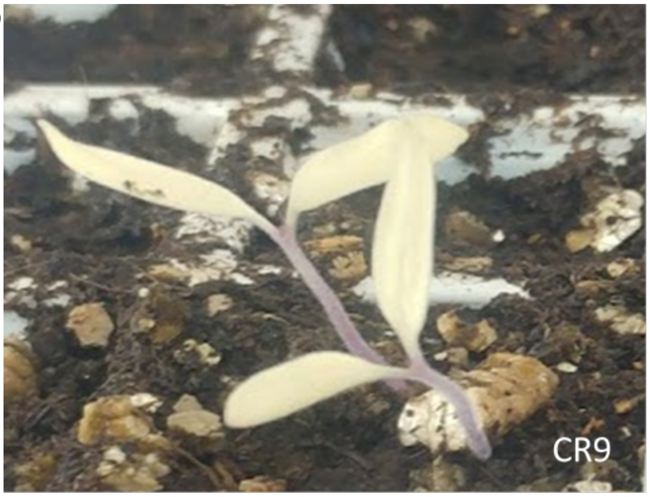

E

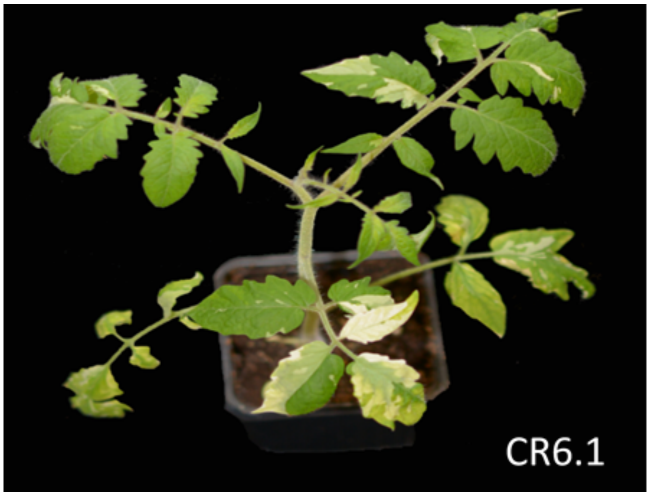

Supplemental Figure 2. CRISPR/Cas9 mutations generated in Solyc01g108200. Validation of the Solyc01g108200 as the gene causing the *canal-1* mutation using the CRISPR/Cas9 system. (A) Scheme and sequence of the CRISP/Cas 9 mutation position CR9, i.e., a deletion of 6 nucleotides, in the first exon of the gene Solyc01g108200. Also shown is the sequence of the guided RNA and the protospacer adjacent motif (PAM) site. (B) Scheme and sequence of mutant CR6.1, which involved deletion and insertion of a nucleotide. (C) The amino acid sequence of M82 and the two mutations (CR6.1 – change of 29 amino acids, CR9 – stop codon after 22 amino acids). (D) Image of CR9 cotyledons; the severity of the mutation did not allow for true leaves to develop. (E) Image of CR6.1 about 30 days after germination. This plant did not produce seeds.

Supplemental Table 1

Introgression Line	Genotype	Mean (CV (%))	Tukey-Kramer's (α=0.05)	Mean (Total Yield (Kg))	Tukey-Kramer's (α=0.05)
IL 1-1	*1*	29.1	B	8.8	A
	2	48.9	B	5.8	A
	3	89.2	A	0.5	B
IL 3-3	*1*	39.6	B	6.3	A
	2	47.4	AB	3.9	B
	3	64.3	A	0.7	C
IL 3-4	*1*	32.6	AB	8.1	A
	2	30.4	B	6.7	A
	3	51.0	A	1.6	B
IL 10-2-2	*1*	32.7	A	6.6	A
	2	21.9	B	8.1	A
	3	31.8	A	7.2	A
IL 12-1-1	*1*	27.7	A	7.5	A
	2	30.1	A	8.0	A
	3	37.1	A	8.0	A

Supplemental Table 1. Summary of field trial Akko 2013. A validation of yield canalization prediction conducted in 3 unstable lines (IL1-1, IL3-3, IL3-4) and two stable lines (IL10-2-2, IL12-1-1). Each of these lines was tested in six canalization replications plots in the spring season of 2013. Mean total yield (TY) and mean coefficient of variation (CV) for each of the ILs tested are shown. Tukey-Kramer's range test was performed for the three genotypic groups of each of the ILs; values followed by the same letter are not significantly different from each other. IL12-1-1 was not as stable as predicted based on the historical data; other lines were stable or not as expected. For each of the ILs genotype *1* represents M82 genome, genotype *2* represent heterozygous, and genotype *3* represent the introgression line homozygous for the wild species segment.

Supplemental Table 2

Trait	Genotype	Mean (CV%)	Tukey-Kramer's (α=0.05)	Mean	Tukey-Kramer's (α=0.05)
1000 Seeds Weight (g)	*1*	7.4	A	3.1	A
	2	6.7	A	3.1	A
	3	7.1	A	3.1	A
Brix (%)	*1*	6.8	A	3.7	A
	2	6.8	A	3.9	A
	3	7.1	A	4.0	A
Plant Weight (Kg)	*1*	30.1	A	2.1	B
	2	28.3	A	2.3	AB
	3	30.4	A	2.4	A
Single Fruit Weight (g)	*1*	12.2	A	71.7	B
	2	11.6	A	74.2	AB
	3	11.6	A	76.4	A
Total Yield (Kg)	*1*	33.8	A	9.6	A
	2	26.1	B	10.6	A
	3	27.4	B	9.4	A

Supplemental Table 2. A comparison of different traits of IL10-2-2. This table gathered the mean value of five traits (1000 seeds weight, brix, plant weight, single fruit weight, total yield) that were measured in the field trials of the three IL10-2-2 genotypes; values followed by the same letter are not significantly different from each other.It can be seen that the mean CV index was significant only for the total yield trait. Genotype *1* represents M82 genome, genotype *2* represent heterozygous, and genotype *3* represents the introgression line 10-2-2.

Supplemental Table 3

Line name	Mutant number	Description	N	Mean CV (Total Yield)
canal-1	e4058	Tomato DNAJ	6	100.7
double feathered	n4596	double feathered	3	98.0
M3	e4936	Thick stem SD	6	93.0
M3	e0112	high yield	5	80.6
M3	e4895	late	6	80.5
M3	e2871	large plant, late	6	73.2
e0137			6	64.3
Clausa	e3545	Clausa	6	60.9
M3	e9003	Black. Long a-symmetrical inflo.	5	58.1
dwarf	e0862	dwarf	6	57.7
N15	n5296	N15	5	56.1
0.0163		0.0163	5	55.7
M3	e4944	SD, nipple	6	53.0
M3	e1297	high HI	6	52.8
0.023		0.023	6	49.9
N2	e4489	N2	4	49.2
La-2/+		La-2/+	5	49.0
M3	e4894	high HI	6	44.3
0.075		0.075	6	44.0
ago7	e4714	ago7	5	42.6
La-6		La-6	6	42.3
N10	e0988	N10	6	42.1
0.093		0.093	6	38.3
dwarf	e4052	dwarf	6	37.7
M3	e4464	S	6	35.8
0.089		0.089	6	35.5
M82			6	34.6
strigo 1		Strigo-1	6	34.3
N5	n1258	N5	5	32.5
N6	e3118	N6	5	32.2
dwarf	e4064+	dwarf	6	31.5
M3	e9181	high yield	6	30.1
N8	e3321	N8	6	30.1
N1	n5568	N1	4	29.9
N7	n1669	N7	6	29.5
N3	e4632	N3	6	29.4
j1, sp- from 2010-111-2		j1,sp-	6	28.5
M3	e0478	late	4	28.1
M3	e4203	high yield, late	6	28.0
N9	n2537	N9	6	27.9
e850			6	27.2
entire	e3335	entire	6	25.4

N4	e4130	N4	7	25.0
M3	e1137	small plant, large fruit	6	24.4
M3	e2674	high HI	6	23.5
M3	e4724	potato leaf	6	23.3
P&P			2	21.0
M3	e9381	small bushy	6	17.1
entire	e2978	entire	5	16.2

Supplemental table 3 – A mutant screen for the yield canalization effect. Forty-eight ethyl methanesulfate (EMS) mutants were screened in a canalization replications plots field trial design. The table is sorted by CV index, from high to low value. e4058 *canal-1* is at the first place, which means it has high environmental sensitivity. M82 is located at the middle of the list. N represents the number of CANAREPs included in the analysis.

Supplemental table 4

IL	HUJI Name	Forward Primer	Reverse Primer	Enzyme	(*S.pennellii* / M82)bps
IL1-1	1-1g48050	TGAAAGAGGAAATAGGAGGATATGAAC	ATCGTGACTGGCGATATATTACGAG	TaqI	750+170 / 920
IL3-3	3-5g12370	AGGGTTGCTGCTGCAGCTGCTTC	TCACCAGCAGCAGCTGCTGCTTC	Hinfl	100+50 / 150
IL3-4	3-5g60160	ACACAATGCTAATCAACGTTATGC	TCATCCACCGCGCACATTTC	PCR	400+50 / 450
IL10-2-2	10-3g54360	AGCTTTCCTGGTTCAACAAGCC	AACTGCTCCGAGCTGTGAGCAC	Hinfl	300+50 / 350
IL12-1-1	12-2g32950	ATATATGCTGTGTAAAATATAATCCTGG	TGGCTGGTGTTCCTCAAGTCATAATAATG	CfoI (Hha I)	450+200 / 650

Supplemental Table 4 – Markers used to validate introgressions in 2013. These markers are part of a set of markers designed to differentiate wild species from the cultivated species. These five markers were used to identify and validate the introgression in the populations tested in the spring season of 2013.

Supplemental table 5

Marker name	Forward sequence	Reverse sequence	(*S.pennellii* / M82)bps
10-2-2 M2	ATATTGAGCACTTTGCATGTGATGA	GTCTCTTTATATGGTGTTGGATAAT	405 / 480
10-2-2 M3	GCAGTTGTTCCAGCAATATTG	GGAAATCTACCTCCACAAGGTAGT	480 / 630
10-2-2 M5	AAGCACATGACTACACCTAAACCAGAT	ATATTTATAGTGGATTGCTCATCTCCTTTG	414 / 590
10-2-2 M6	AAGCAAATTTATACCACTAGACAGG	GACTAGACGAGAATCTTATCCTAATCAGTG	475 / 412
10-2-2 M8	TTATGCCGACAGAAAGAGACAGTT	TTAAGGGCTTTGAGAGTTTTGGTT	475 / 672

Supplemental Table 5 –Markers developed to differentiate between the different recombinants of IL10-2-2. These markers are based on indels between the genomes of the wild species *S. pennellii* (LA0716) and the cultivated inbred variety *S. lycopersicum* cv.M82.
